# Energy-Efficient Deadline-Aware Data-Gathering Scheme Using Multiple Mobile Data Collectors

**DOI:** 10.3390/s17040742

**Published:** 2017-04-01

**Authors:** Rumpa Dasgupta, Seokhoon Yoon

**Affiliations:** Department of Electrical and Computer Engineering, University of Ulsan, Ulsan 680-749, Korea; rumpa.dasgupta3949@gmail.com

**Keywords:** multiple mobile data collectors, data-gathering, energy constraint, delay constraint, wireless power transfer

## Abstract

In wireless sensor networks, the data collected by sensors are usually forwarded to the sink through multi-hop forwarding. However, multi-hop forwarding can be inefficient due to the energy hole problem and high communications overhead. Moreover, when the monitored area is large and the number of sensors is small, sensors cannot send the data via multi-hop forwarding due to the lack of network connectivity. In order to address those problems of multi-hop forwarding, in this paper, we consider a data collection scheme that uses mobile data collectors (MDCs), which visit sensors and collect data from them. Due to the recent breakthroughs in wireless power transfer technology, MDCs can also be used to recharge the sensors to keep them from draining their energy. In MDC-based data-gathering schemes, a big challenge is how to find the MDCs’ traveling paths in a balanced way, such that their energy consumption is minimized and the packet-delay constraint is satisfied. Therefore, in this paper, we aim at finding the MDCs’ paths, taking energy efficiency and delay constraints into account. We first define an optimization problem, named the delay-constrained energy minimization (DCEM) problem, to find the paths for MDCs. An integer linear programming problem is formulated to find the optimal solution. We also propose a two-phase path-selection algorithm to efficiently solve the DCEM problem. Simulations are performed to compare the performance of the proposed algorithms with two heuristics algorithms for the vehicle routing problem under various scenarios. The simulation results show that the proposed algorithms can outperform existing algorithms in terms of energy efficiency and packet delay.

## 1. Introduction

Wireless sensor networks (WSNs) comprise sensor nodes that are deployed to sense physical or environmental conditions for a diverse range of applications, including environmental monitoring, industrial sensing, battlefield surveillance, critical response, and so forth [1]. Traditionally, the sensing data are forwarded to a sink via multiple hops. However, it is known that multi-hop forwarding leads to the energy-hole problem, where sensors near the sink consume much more energy, since they forward more data packets than sensors farther from the sink [2]. Once these sensors run out of battery power, other sensors can no longer reach the sink, and the network becomes disconnected and decreases overall network lifetime. Moreover, multi-hop forwarding may incur high communications overhead due to frequent information exchange among sensors, and data transmission is affected by interference and collisions [3]. As a result, packet loss increases along with increases in the number of hops.

To avoid these problems, the mobile data-gathering scheme has been explored in recent years [4,5,6,7,8,9,10,11,12,13,14,15,16]. In such schemes, mobile data collectors (MDCs) (e.g., mobile sensors, robotic vehicles) roam in the network, collect data from sensors via short-range communications and bring the data to the sink. By using MDCs for data collection, reliability in data delivery can be achieved, and energy consumption in transmitting the data can be reduced significantly. However, MDC-based data-gathering schemes can only slow down energy depletion of sensors, rather than prevent it.

In this paper, we consider using wireless power transfer technology to keep the WSN operational without the risk of energy depletion. The wireless power transfer technology is mainly classified into two categories: far-field [17,18,19,20,21,22] and near-field [23,24,25]. In the far-field or radiative technique, power is transferred by beams of electromagnetic radiation. Recently, this technique has been used in stationary wireless sensors networks in order to transfer power to sensor nodes [19,20,21,22]. In the near-field technique, power is transferred by magnetic fields or by electric fields. For example, in [24], Kurs et al. showed that wireless power transfer is feasible by using a technique called magnetic resonant coupling, i.e., electric power can be transferred from one storage device to another device without wires. The near-field technique has high power transfer efficiency at a shorter distance. In this work, the near-field technique is considered, since we can exploit the mobility of MDCs, which can approach the sensors and achieve high energy transfer efficiency, i.e., MDCs can recharge sensors to a certain energy level when they visit them periodically, such that the WSN remains operational without the risk of energy depletion.

In MDC-based data-gathering schemes, minimizing movement energy is critical, since MDCs consume most of their energy for movements. Balancing energy consumption is also important in the case of multiple MDCs. For example, some MDCs may travel a longer distance, compared to others, which results in unbalanced energy consumption and being unable to return to the sink. Meanwhile, guaranteeing packet delivery delays is also crucial for achieving acceptable quality of service in some applications. For example, the detection of critical events (e.g., intruder detection, forest fire detection [26]) should be reported to the sink within a predetermined time interval.

There have been many studies on data gathering in WSNs using MDCs [4,5,6,7,8,9,10,11,12,13,14,15,16]. In addition, some studies focused on energy replenishment in WSNs using MDCs [25,27,28,29]. However, most of the previous studies did not consider important factors, such as the MDCs’ energy consumption for movement, their limited battery capacity, their travel time and the recharging time of the sensors. Moreover, none of them considered minimizing total energy, in cases of multiple MDCs, while meeting packet delivery delay requirements.

Therefore, in this work, we focus on finding the MDCs’ paths that minimize total energy consumption while satisfying delay and energy constraints. We first formulate an integer linear programming (ILP) optimization problem. The objective of ILP formulation is to find the paths for MDCs that minimize the total energy consumption while guaranteeing that packets should reach the sink within a given deadline, and in each period, the energy consumption of each MDC (including energy for wireless charging, moving and data reception) cannot exceed its energy capacity limit; and every sensor should have residual energy above a minimum threshold at all times.

We note that finding the optimal paths for MDCs using the ILP is computationally expensive, particularly when the number of MDCs or the number of sensors is large. Therefore, in this paper, we propose efficient path selection algorithms that have two phases: clustering and group membership adjustment. We propose two clustering algorithms, angle-based clustering (ABC) and angle-based clustering with path-length ratio (ABC-PR), for partitioning the sensors into multiple groups. Then, two group-membership adjustment algorithms, nearest node assignment (NNA) and node assignment with maximal path-length decrease (MPD), are proposed to minimize the energy consumption of the MDCs and meet constraints, including the packet delay and residual energy of the sensors.

In the proposed clustering algorithms, nodes are partitioned into different groups in such a way that the travel distance of each MDC is close to that of the other MDCs. Then, in the group membership-adjustment algorithms, the group that has the maximum travel distance is selected. From this group, one node is reassigned to its clockwise adjacent group. Specifically, when finding a node for reassignment, NNA selects the node that is the nearest to its clockwise adjacent group, and MPD selects the node whose membership change reduces the path length the most.

Simulations and analysis are conducted to evaluate the performance of the proposed algorithms in various scenarios. The performance of the proposed algorithms is compared with two well-known heuristic algorithms for the vehicle routing problem (VRP) and VRP variants. Simulation results show that the proposed algorithms outperform existing approaches in terms of energy consumption and delay. Simulation results also indicate that MPD with ABC-PR shows the best performance, compared to others, and achieves a near-optimal solution.

The rest of the paper is organized as follows. In Section 2, we present an overview of existing studies related to our work. Section 3 introduces general assumptions and the network model considered in this study. In Section 4, the problem definition and problem formulation are presented. In Section 5, we describe the proposed two-phase path-selection algorithm. Section 6 presents the setup for simulation and performance analysis. Finally, we conclude the paper in Section 7.

## 2. Related Work

In this section, we present some existing studies that considered mobile data collectors for data collection and recharging sensor nodes in WSNs and then compare those studies with our work.

Many recent studies have been conducted for data collection in WSNs using a single MDC with different objectives [4,5,6,7,8,9,10,11,12]. For instance, three studies [6,7,8] focused on the minimization of packet delay with meeting a given deadline. Specifically, Xing et al. [6] proposed a rendezvous-based data collection scheme in which a group of nodes acts as rendezvous points to buffer and collect data received from source nodes and deliver them to a mobile base station within a given time period. In [7], Kansal et al. described a problem that assumes an MDC must traverse a given path within a predetermined time interval, with the objective of controlling the speed of the MDC in order to maximize data collection. A heuristic algorithm was proposed by Sugihara and Gupta [8], which considers both timing constraints and sensor locations to reduce packet delay in a data mule scheduling problem.

Somasundara et al. [9] and Gu et al. [10] studied the scheduling problem of MDC to avoid data loss at sensors due to buffer overflow. For instance, in [10], Gu et al. first partitioned the sensor nodes into multiple groups, depending on their locations and data-generation rates. Then, in each group, the MDC is scheduled to visit stationary sensors at a sufficient frequency to minimize buffer overflow.

In addition, Wang et al. [11] focused on the problem of maximizing network lifetime. They considered a mobile sink for visiting some sensor nodes in the network and formulated a linear programming optimization problem to determine the optimal path for the mobile sink in order to maximize network lifetime.

However, those works use a single MDC to collect data from the entire area. The MDC will take a long time to finish one tour when the size of the area is large. As a result, packet delay increases. Thus, only one MDC may not be useful for delay-sensitive applications. To minimize packet delay, this work uses multiple MDCs to collect data from sensors.

There are a number of studies that use multiple MDCs for data collection in WSNs [13,14,15,16]. For example, Keung et al. [14] and Aslanyan et al. [15] considered multiple MDCs for data collection from source nodes. They assumed that MDCs have random mobility patterns. Because the MDCs have random mobility, those schemes cannot estimate packet delay or the travel time of MDCs. Jea et al. also proposed a multiple MDC-based data collection scheme [16]. The authors considered MDCs that follow parallel straight lines for their movement and that collect data from sensors via multiple hops. This scheme is suitable for uniformly-distributed sensor networks. However, that type of MDC movement is not practical, because different obstacles may block the paths of the MDCs. In addition, all of the listed studies considered MDCs only for data collection.

In contrast, our work considers multiple MDCs that not only collect data from the sensors, but also replenish the energy in the sensors to keep the WSN operational without the risk of energy depletion. Moreover, we focus on finding MDCs’ paths that minimize total energy consumption, with delay and energy constraints for each MDC.

There have been some studies that use MDCs to recharge sensor nodes’ batteries wirelessly via wireless power transfer [25,27]. In those works, the collected data are forwarded to the sink through multiple hops. For example, in [25], Xie et al. considered a mobile charging vehicle that periodically recharges the sensor nodes’ batteries to keep the energy level of each sensor above a minimum threshold. Peng et al. [27] developed a proof-of-concept prototype, and experiments were performed on the prototype to estimate its energy replenishment performance in small-scale networks. Although sensors’ energy was replenished in those works, a significant amount of sensor energy was still wasted due to the multi-hop data transmissions. In our work, MDCs are used for energy replenishment, as well as data collection when visiting sensors, bringing the collected data to the sink within a predetermined time interval.

There are few works that consider MDCs for both collection and energy replenishment in WSNs [28,29]. For example, Pan et al. [28] studied an energy replenishment and data collection problem considering wireless charging, flow routing and data transmission constraints to determine the specific sensors, which need to be recharged using a wireless charging vehicle, so that network lifetime is maximized. However, those works did not consider delay requirements, which is important for achieving an acceptable quality of service. In addition, travel time of an MDC, the time consumed for recharging sensors and the MDC’s moving energy consumption were neglected in those works.

In contrast, we consider every possible energy consumption of the MDCs, which includes MDCs’ movement energy, energy consumption for receiving data from sensors and recharging the sensors. Moreover, travel time and sojourn time of the MDCs at each sensor node are considered in our work.

There exist some similarities between our work and the vehicle routing problem (VRP) [30] with its variants. The VRP is defined as finding routes for vehicles to deliver goods between a depot and customers to minimize the vehicles’ total travel cost. Among different variants of VRP, our work is related to VRP with time windows (VRPTW) [31]. In VRPTW, vehicles are scheduled to visit all customers within a pre-defined time window. On the other hand, in this study, we consider both energy and timing constraints for each MDC. We also compare the performance of our proposed algorithms with two heuristic algorithms for VRP.

## 3. Network Model

**Figure 1:**
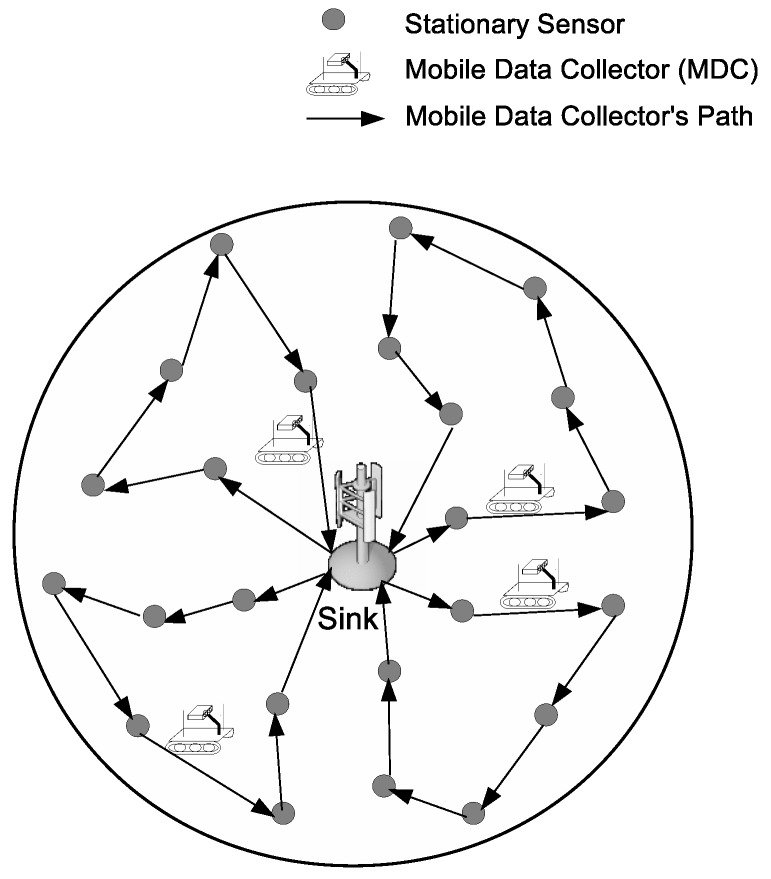
Network Model.

In this section, we present the network model of the proposed scheme in detail. Figure 1 illustrates the network model of our proposed scheme. The considered network consists of a number of stationary sensors and a number of mobile data collectors. Sensors are deployed randomly in a circular area around the sink, with radius *R*, and MDCs are used to collect data from sensors and to deliver the data to the sink. In this paper, we denote *N* and *K* as the total numbers of sensors and MDCs, respectively. It is assumed that the sensor positions are known a priori. Each sensor periodically generates data and saves them in its buffer until an MDC visits it and collects the data. Besides, we assume that the generated data over the WSN should periodically be delivered to the sink within time interval *D*. Thus, each MDC needs to finish its tour within the deadline, *D*. Sensors are provided with a limited battery and consume energy for operation. Thus, the WSN can only remain operational for a limited amount of time. To keep the sensors from completely draining their energy and to keep the WSN alive, MDCs periodically recharge the visited sensors to a certain energy level. Each sensor node (except the sink) is visited by only one MDC. Each MDC starts its journey at the sink, and its speed is *v*. When it arrives at a sensor node, say sensor *i*, it will spend time $t_{i}$ to charge the sensor node’s battery wirelessly via wireless power transfer [24] and collects data from this sensor. After $t_{i}$, the MDC leaves sensor *i* and travels to the next sensor on its path. Moreover, let *Q* denote the battery capacity of each MDC, which is fully charged at the beginning of the tour. When the MDC finishes visiting sensors, it brings the collected data back to the sink. After that, the MDC battery will be replaced [25] to get it ready for the next tour with a fully-charged battery.

In this work, the energy model is the one referred to by Liu et al. [32]. Let $e_{S}$ denote the energy consumption for sensing one bit of data. Furthermore, $e_{T}$ and $e_{R}$ denote the energy usage per bit of transmission and reception electronics, respectively. The energy consumed by a transmission amplifier for transmitting one bit is ρ $l^{\gamma }$, where γ is the signal decline factor, and *l* is the distance between transmitting and receiving nodes. Transmission energy $e_{tx}$ and reception energy $e_{rx}$ for one bit of data can be calculated as:

(1) $$\begin{array}{c} e_{tx}=e_{T}+\rho l^{\gamma }\\ e_{rx}=e_{R} \end{array}$$

## 4. Problem Definition and Formulation

From the given description of the considered network and corresponding model, in this section, we define and formulate our problem.

### 4.1. Problem Definition

This work aims at finding paths for MDCs to minimize the total energy consumption while satisfying delay and energy constraints. The problem of finding paths can be formally defined as follows.

**Definition** **1.** Delay-constrained energy minimization (DCEM): Find the MDCs’ paths that minimize the total energy consumption of all MDCs with the delay constraints that packets should reach the sink within a given deadline, D, the energy consumption of each MDC cannot exceed its energy capacity, Q, in each period, and every sensor should have at least the minimum residual energy, $e_{min}$, all of the time.

Note that DCEM is a more generalized problem than VRP [30]. Since the VRP is NP-hard [30], DCEM is also NP-hard.

### 4.2. Problem Formulation

Now, we present an integer linear programming (ILP) formulation for DCEM. Definitions for symbols used in the ILP formulation are presented in Table 1.

Let us assume that a network consists of a set of nodes, V={1,…,N}, where the element 1 represents the sink, and a set of arcs, E={(i,j):i,j∈V,i≠j}. Furthermore, let us define *M* as a set of MDCs where M={1,…,K}.

Let $c_{ij}$ denote the travel distance (Euclidean distance) from node *i* to node *j*. We also define $x_{ij}^{k}$ as a binary decision variable that becomes one if arc(i,j) is on the paths of the MDC *k*, k∈M, but becomes zero otherwise. That is,

(2) $$x_{ij}^{k}=\left\{\begin{array}{cc} 1 & \mathrm{if}\;arc(i,j)\;\mathrm{is}\;\mathrm{on}\;\mathrm{path}\;\mathrm{of}\;\mathrm{MDC}\;k.\;i,j\in V,\;k\in M\\ 0 & o/w \end{array}\right.$$

Furthermore, $u_{i}^{k}$ is defined as the number of nodes visited by MDC *k* up to node *i*. We also define $t_{ij}$ as the travel time between node *i* and node *j*. Recall that $t_{i}$ is the sojourn time of the MDC at node *i* for charging and collecting data. Note that $t_{1}$ represents the sojourn time of the MDC at the sink. Each MDC spends a small amount of time, $t_{1}$, at the sink to replace its own battery. $T_{k}$ refers to the time for one round journey of MDC *k* or the time for one period. Therefore, $T_{k}$ can be calculated as follows:

(3) $$T_{k}=\;\sum_{i=1}^{N}\sum_{j=1}^{N}x_{ij}^{k}\left(t_{ij}+t_{i}\right)-t_{1},\;k=1,\ldots ,K$$

Let $E_{m}$ denote energy consumption by the MDC to travel one meter, and *r* is the data-generation rate of a sensor (in bits per s). Furthermore, $E_{i}^{ch}$ denotes the energy transfer rate from the MDC to sensor *i*. Recall that $e_{rx}$ is the energy consumption to receive one bit of data. We define $E_{Q}^{k}$, $E_{Mv}^{k}$ and $E_{rx}^{k}$, respectively, as energy consumed by MDC *k* for wireless charging of visited sensors, its movement energy and the energy consumption to receive data from visited sensors during its one-round journey. Therefore, in each period, the total energy consumption of MDC *k* (k∈M) can be calculated as follows:

(4) $$\begin{array}{c} E_{Q}^{k}+E_{Mv}^{k}+E_{rx}^{k}=E_{i}^{ch}t_{i}\sum_{i=1}^{N}\sum_{j=2}^{N}x_{ij}^{k}+E_{m}\sum_{i=1}^{N}\sum_{j=1}^{N}x_{ij}^{k}c_{ij}+\;re_{rx}D\sum_{i=1}^{N}\sum_{j=2}^{N}x_{ij}^{k} \end{array}$$

Each sensor consumes energy for generating data and transmitting buffered data to the MDC. Recall that $e_{tx}$ and $e_{S}$ represent the consumed energy for transmitting and generating one bit of data, respectively. Furthermore, let η denote the energy transfer efficiency between the MDC and sensors. Then, after generating the data, transmitting them to the MDC and energy replenishment, the residual energy at sensor *i* by the end of a period is:

(5) $$\;E_{i}^{ch}\eta t_{i}-rT_{k}e_{S}-rT_{k}e_{tx},\;i=2,\ldots ,N$$

We replace $T_{k}$ from Equation (3); then, Equation (5) can be rewritten as follows:

(6) $$\begin{array}{c} E_{i}^{ch}\eta t_{i}-r\;(\sum_{i=1}^{N}\sum_{j=1}^{N}x_{ij}^{k}\left(t_{ij}+t_{i}\right)-t_{1})e_{S}-r\;(\sum_{i=1}^{N}\sum_{j=1}^{N}x_{ij}^{k}\left(t_{ij}+t_{i}\right)-t_{1})e_{tx},\;i=2,\ldots ,N \end{array}$$

Now, the ILP problem can be formulated as follows:

(7) $$\begin{array}{c} \mathrm{minimize}\;E_{i}^{ch}t_{i}\sum_{k=1}^{K}\sum_{i=1}^{N}\sum_{j=2}^{N}x_{ij}^{k}+E_{m}\sum_{k=1}^{K}\sum_{i=1}^{N}\sum_{j=1}^{N}x_{ij}^{k}c_{ij}+re_{rx}D\sum_{k=1}^{K}\sum_{i=1}^{N}\sum_{j=2}^{N}x_{ij}^{k} \end{array}$$

(8) $$\begin{array}{c} \mathrm{subject}\;\mathrm{to}\;\sum_{i=1}^{N}\sum_{k=1}^{K}x_{ij}^{k}=1,\;j\in V\setminus \left\{1\right\} \end{array}$$

(9) $$\begin{array}{c} \;\;\sum_{j=1}^{N}\sum_{k=1}^{K}x_{ij}^{k}=1,\;i\in V\setminus \left\{1\right\} \end{array}$$

(10) $$\begin{array}{c} \;\sum_{i=1}^{N}x_{ip}^{k}-\;\sum_{j=1}^{N}x_{pj}^{k}=0,\;k\in M,\;p\in V\setminus \left\{1\right\} \end{array}$$

(11) $$\begin{array}{c} \;\sum_{j=2}^{N}x_{1j}^{k}=1,\;k\in M \end{array}$$

(12) $$\begin{array}{c} \;\sum_{i=2}^{N}x_{i1}^{k}=1,\;k\in M \end{array}$$

(13) $$\begin{array}{c} \;\sum_{i=1}^{N}\sum_{j=1}^{N}x_{ij}^{k}\left(t_{ij}+t_{i}\right)-t_{1}\leq D,\;k\in M \end{array}$$

(14) $$\begin{array}{c} E_{i}^{ch}t_{i}\sum_{i=1}^{N}\sum_{j=2}^{N}x_{ij}^{k}+E_{m}\sum_{i=1}^{N}\sum_{j=1}^{N}x_{ij}^{k}c_{ij}+\;re_{rx}D\sum_{i=1}^{N}\sum_{j=2}^{N}x_{ij}^{k}\leq Q,\;\;k\in M \end{array}$$

(15) $$\begin{array}{c} E_{i}^{ch}\eta t_{i}-r\;(\sum_{i=1}^{N}\sum_{j=1}^{N}x_{ij}^{k}\left(t_{ij}+t_{i}\right)-t_{1})e_{s}-r\;(\sum_{i=1}^{N}\sum_{j=1}^{N}x_{ij}^{k}\left(t_{ij}+t_{i}\right)-t_{1})e_{tx}\geq e_{min},\;i\in V\setminus \left\{1\right\} \end{array}$$

(16) $$\begin{array}{c} \;u_{1}^{k}=1,\;k\in M \end{array}$$

(17) $$\begin{array}{c} \;u_{i}^{k}-u_{j}^{k}+1\leq \left(N-1\right)\left(1-x_{ij}^{k}\right),\;k\in M,\;i,j\in V\setminus \left\{1\right\} \end{array}$$

The objective function for the formulation is represented by Equation (7). Recall that every MDC consumes energy for wireless charging of visited sensors, its movement and receiving data from visited sensors. In Equation (7), the first, second and third term represent the energy consumption by all MDCs for wireless charging, moving and data reception, respectively. Therefore, Equation (7) presents the total energy consumption of all MDCs. The objective is to minimize the total energy consumption by finding optimal paths of MDCs.

Equations (8)–(17) represent constraints on the paths of the MDCs. More specifically, Constraints (8) and (9) ensure that every node except the sink is visited exactly once by only one MDC. For example, if MDC *k* arrives at node *j* (j∈V∖{1}) from node *i*, then $x_{ij}^{k}=1$. Since $\sum_{i=1}^{N}\sum_{k=1}^{K}x_{ij}^{k}=1$, other MDCs from any other node cannot visit node *j*. Furthermore, when MDC *k* leaves node *j*, it only goes to one node among the *N* nodes to satisfy Constraint (9).

Constraint (10) is the route continuity constraint, which ensures that once an MDC arrives at a node, it must also leave that node. Specifically, if MDC *k* arrives at a node, say node *p* from node *i*, then $x_{ip}^{k}=1$. Constraint (10) is satisfied whenever $x_{pj}^{k}=1$, i.e., MDC *k* leaves node *p* and travels to another node *j*. Constraint (11) ensures that MDC *k* starts its tour from the sink to any other node, say node *j* (i.e.,$\sum_{j=2}^{N}x_{1j}^{k}=1$), and Constraint (12) ensures that MDC *k* returns to the sink from exactly one node among the *N* nodes (i.e.,$\sum_{i=2}^{N}x_{i1}^{k}=1$). Recall that 1 represents the sink.

Constraint (13) is the delay constraint for each MDC. It ensures that time for one round journey of MDC *k* (k∈M), which includes the travel and charge time of the MDC, cannot exceed the given deadline, *D*. The time MDC *k* needs to finish a one-round journey is estimated in Equation (3). Constraint (14) limits the maximum energy consumption of each MDC. More specifically, it ensures that energy consumption by every MDC, which includes wireless charging, moving and data reception, cannot exceed its energy capacity, *Q*. The total energy consumption of MDC *k* (k∈M) is calculated in Equation (4). Constraint (15) ensures that at the end of the current period, every sensor has the minimum residual energy, $e_{min}$, after being recharged by the MDC and consuming energy for generating and transmitting data. The residual energy of sensor *i* (i∈V∖{1}) at the end of a period is presented in Equation (5).

Constraints (16) and (17) eliminate the sub-tours in the paths of the MDCs in a way similar to Miller–Tucker–Zemlin (MTZ) sub-tour elimination of the traveling salesman problem (TSP) [33]. MTZ sub-tour elimination constraints eliminate a collection of the disjoint sub-tour that together satisfies Constraints (8)–(15). Recall that $u_{i}^{k}$ is the number of nodes visited by MDC *k* up to node *i*. Constraint (16) sets the value of $u_{i}^{k}$ for the sink (i=1) to be one, i.e., $u_{1}^{k}=1$, and Constraint (17) ensures that $u_{j}^{k}\geq u_{i}^{k}+1$, when $x_{ij}^{k}=1$. Thus, Constraints (16) and (17) together prohibit the formation of any sub-tour within nodes in V∖{1}.

### 4.3. Alternative Objectives

In this work, we focus on minimizing the total energy consumption. Depending on the requirements, alternative objectives can be considered. For example, the travel distance can be a main concern in some cases. Then, the problem can be defined as minimizing the total distance traveled by all MDCs and satisfying the delay and energy constraints. That is,

(18) $$\mathrm{minimize}\;\sum_{i=1}^{N}\;\sum_{j=1}^{N}\sum_{k=1}^{K}c_{ij}x_{ij}^{k}$$

Meanwhile, in some applications, mainly packet delay needs to be considered. In such a case, the objective can be minimizing the maximum tour time of the MDCs. Since data delivery relies on the physical movement of the MDCs, packet delay can be reduced by minimizing their tour time. Let τ denote the maximum time from among the MDCs to finish one round journey. Thus, the problem can also be expressed as follows: minimize the maximum tour time of MDCs, τ, while satisfying the delay and energy constraints. That is,

(19) minimizeτ

(20) $$\begin{array}{c} \mathrm{subject}\;\mathrm{to}\;\;\sum_{i=1}^{N}\sum_{j=1}^{N}x_{ij}^{k}\left(t_{ij}+t_{i}\right)-t_{1}\leq \tau ,\;k\in M \end{array}$$

(21) τ≤D

In this case, delay Constraint (13) is replaced with Constraints (20) and (21). More specifically, Constraint (20) ensures that the travel time and charging time of every MDC cannot exceed the maximum tour time, τ. Constraint (21) ensures that the maximum time an MDC needs to finish a one-round journey cannot exceed the given deadline, *D*.

## 5. Path Selection Algorithm with Delay and Energy Constraints

To find the optimal solution for the DCEM problem, the proposed ILP formulation can be solved using available optimization tools. However, it requires highly expensive computation and a long execution time when the number of MDCs or the number of sensors is large. Therefore, in this work, we propose efficient two-phase path-selection algorithms with delay and energy constraints. In Phase 1, clustering is performed to partition the sensors into several groups, and in Phase 2, the membership of each group is adjusted in order to meet the constraints and minimize the travel distances of MDCs. This paper proposes two clustering algorithms, angle-based clustering (ABC) and angle-based clustering with path-length ratio (ABC-PR). Then, two group membership-adjustment algorithms, nearest node assignment (NNA) and node assignment with maximal path-length decrease (MPD), are proposed. We first present the clustering algorithms. Then, two group membership-adjustment algorithms are described.

### 5.1. Clustering Algorithms

In our scheme, the sensor nodes are partitioned into *K* groups, where *K* is the number of MDCs used in the network. The generated data should be delivered to the sink within a given deadline, *D*, and the energy consumption of each MDC cannot exceed its energy capacity, *Q*. Thus, each MDC needs to finish its tour within delay constraint *D* and energy constraint *Q*. In order to satisfy the deadlines, it is desirable for MDCs to have a tour where the length is close to other MDCs, so that each MDC can meet the deadlines. Therefore, we propose the ABC and ABC-PR algorithms. The objective of ABC and ABC-PR is to divide the sensor nodes into *K* groups such that the travel distance of each MDC is close to the distance of the other MDCs.

#### 5.1.1. Angle-Based Clustering

The main idea of the ABC algorithm is simple. ABC first divides the entire circular area into *K* sectors with equal angles, and the nodes located in the same sector belong to the same group. Then, the group (sector) angle is increased or decreased to transfer some nodes from one group to another. This process is repeated between two adjacent groups in a cyclic pattern in order to balance the travel distances of the MDCs with each other.

**Figure 2:**
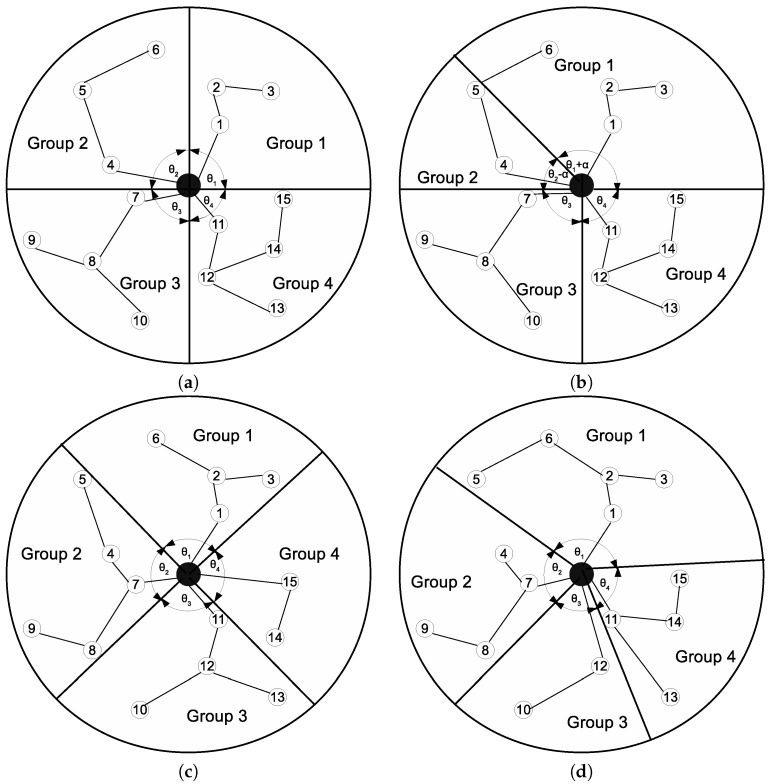
An example of angle-based clustering: (**a**) initial partitioning of the area and the minimum spanning tree (MST) of the sensors; (**b**) α rotation between Group 1 and Group 2; (**c**) nodes in the four groups after the first iteration; and (**d**) the final node groups.

Figure 2 illustrates node grouping based on ABC. In Figure 2, the sink is located at the center of the area. Let $\theta_{i}$ denote the angle of group *i*, (i=1,…,K), and assume there are four MDCs, i.e., *K* = 4. Therefore, in the example in Figure 2a, the area is divided into four sectors such that $\theta_{1}$ = $\theta_{2}$ = $\theta_{3}$ = $\theta_{4}$. The nodes located in the same sector are considered as the same group members, and the sink is included in every group. In each group, a minimum spanning tree (MST) is built over the nodes, where each tree is rooted at the sink, as shown in Figure 2a. Then, the length for the MST of each group is estimated. Let us define $L_{i}$ and $L_{avg}$ as the length of the MST of group *i*(i=1,…,K) and the average length of the MSTs in all groups, respectively. Furthermore, ζ represents a small positive constant. If the difference between $L_{avg}$ and $L_{i}$ (i=1,…,K) is less than or equal to ζ, we obtain the nodes for *K* groups. Otherwise, the MST length of the two groups is compared cyclically. Specifically, ABC compares the MST length of group *i* with i+1, group i+1 with i+2 and group *K* with *i*. Let α denote a rotation angle, and $\alpha <\frac{\pi }{K}$. In every comparison, if $L_{i}>L_{i+1}$, $\theta_{i}$ is decreased by α and $\theta_{i+1}$ is increased by α. Otherwise, it is vice versa.

In the example in Figure 2a, $L_{1}<L_{2}$. Therefore, $\theta_{1}$ is increased by rotation angle α, and $\theta_{2}$ is decreased by α, as shown in Figure 2b. As a result, Node 6 becomes a member of Group 1 instead of Group 2. The MST lengths for both groups are newly estimated with the addition of Node 6 in Group 1 and the exclusion of Node 6 from Group 2. After one iteration, nodes in the four groups are shown in Figure 2c. The new $L_{avg}$ is estimated, and the difference between $L_{avg}$ and $L_{i}$ (i=1,…,K) is examined. If the difference is greater than ζ, the next iteration begins. Let β denote a positive constant, and β<1. For the next iteration, α is set to βα, and the same process is applied. This process is repeated until the difference between $L_{avg}$ and $L_{i}$ is either less than or equal to ζ or α is less than or equal to the threshold value, ε, where ε≤1. Figure 2d shows the final node groups obtained from ABC. The clustering algorithm is presented in Algorithm 1 (see Appendix A for the detailed clustering algorithm). In Algorithm 1, when ABC is used, ω is obtained from Algorithm 2.

**Algorithm 1** Clustering algorithm.**Input: V,M,α,ζ,β***V*: Set of sensors▹V={1,2,...,N}*M*: Set of groups or MDCs▹M={1,2,...,K}α: Rotation angle▹$\alpha <\frac{\pi }{K}$ζ: Small positive constant▹ζ≥1β: Positive constant▹0<β<1**Output: $fin\Omega_{K}=\left\{N_{1},N_{2},...,N_{K}\right\}$**▹ Set of nodes for *K* groups1:Initialization;2:$N_{i}$ = Set of nodes of group *i*, i∈M;3:Divide the area into K sectors where $\theta_{1}$ = $\theta_{2}$ =…= $\theta_{K}$ = $\frac{2\pi }{K}$ and $cur\Omega_{K}=\left\{N_{1},N_{2},...,N_{K}\right\}$;4:For i=1,…,K, calculate minimum spanning tree (MST) length $L_{i}$ and average length of all MSTs $L_{avg}$;5:**if** $|L_{i}-L_{avg}|>\zeta$ **then**                         ▹ for i∈M6:    **for** each $\left(G_{i}\%K\right)$ group and $\left(G_{i+1}\%K\right)$ group **do**7:        use ABC or ABC-PR to update ω;8:        update $\theta_{i}$ and $\theta_{i+1}$ to $\theta_{i}-\omega$ and $\theta_{i+1}+\omega$, respectively;9:        update $L_{i}$ and $L_{i+1}$ using $N_{i},N_{i+1},\theta_{i},\theta_{i+1}$;10:    **end for**11:**end if**12:Calculate new $L_{avg}$;13:**if** $|L_{i}-L_{avg}|>\zeta$ **then**                        ▹ for i∈M14:    update α to βα;15:    goto 6;16:**else**17:    $fin\Omega_{K}$ = $cur\Omega_{K}$;18:    exit;19:**end if**

**Algorithm 2** Angle-based clustering (ABC).1:**function** ABC($L_{i}$,$L_{i+1}$,α)2:    **if** $L_{i}>L_{i+1}$ **then**3:        ω=α;4:    **else if** $L_{i}<L_{i+1}$ **then**5:        ω=−α;6:    **else**7:        ω=0;8:    **end if**9:    **return** ω10:**end function**

#### 5.1.2. Angle-Based Clustering with Path-Length Ratio

In ABC, $\theta_{i}$ is increased or decreased by rotation angle α, which remains the same for one iteration. For the second iteration, α is set to βα, and so on. Note that in ABC, the value of the rotation angle does not reflect the path lengths of the groups. As a result, when the ABC algorithm is used, travel distance for MDCs remains unbalanced, and an MDC may travel a longer distance, compared to the others. In order to address the problem of ABC, ABC-PR first estimates the path-length ratio to determine how much angular rotation is needed to balance the travel distances of the MDCs between two groups. After that, actual rotation angle $\alpha^{\prime }$ is calculated based on the path-length ratio and rotation angle α. More specifically, in the MST length comparison between groups *i* and i+1, actual rotation angle $\alpha^{\prime }$ is set to $\frac{\max(L_{i},L_{i+1})}{L_{i}+L_{i+1}}\alpha$. When $L_{i}>L_{i+1}$, ABC-PR decreases $\alpha^{\prime }$ from $\theta_{i}$, and increases $\alpha^{\prime }$ to $\theta_{i+1}$. On the other hand, if $L_{i}<L_{i+1}$, $\theta_{i}$ and $\theta_{i+1}$ are increased and decreased by $\alpha^{\prime }$, respectively. The estimation of $\alpha^{\prime }$ in ABC-PR is presented in Algorithm 3.

Now, we estimate the time complexity of the clustering algorithm for one iteration. In the clustering algorithms, the MST lengths of node groups are estimated. The maximum number of nodes in each group is *N*, and the time complexity for finding MST is $O(N^{2})$. The number of calculations for the rest is constant. Therefore, the time complexity of the clustering algorithms can be expressed as $O(N^{2})$.

**Algorithm 3** Angle-based clustering with path-length ratio (ABC-PR).1:**function** ABCPR($L_{i}$,$L_{i+1}$,α)2:    $\alpha^{\prime }=\frac{\max(L_{i},L_{i+1})}{L_{i}+L_{i+1}}\alpha$                 ▹ Calculate $\alpha^{\prime }$3:    **if** $L_{i}>L_{i+1}$ **then**4:        $\omega =\alpha^{\prime };$5:    **else if** $L_{i}<L_{i+1}$ **then**6:        $\omega =-\alpha^{\prime };$7:    **else**8:        ω=0;9:    **end if**10:    **return** ω11:**end function**

### 5.2. Group Membership-Adjustment Algorithms

We obtained *K* sensor groups from the clustering algorithms, and each MDC is assigned to a group for collecting data and charging the sensor nodes’ batteries. In order to meet the constraints of each MDC and minimize the total travel distance of the MDCs, we propose two group membership-adjustment algorithms: nearest node assignment and node assignment with maximal path-length decrease.

#### 5.2.1. Nearest Node Assignment

In the NNA algorithm, an MDC’s path for a group is first determined by the twice around the tree heuristic algorithm of TSP [34]. The borderline between a group and its clockwise adjacent group is defined as the slant. NNA selects the group that has the maximum travel distance among all of the groups. From this group, the node that is nearest to the slant is assigned to its clockwise adjacent group in order to reduce the travel distance of the group with maximum travel distance. The process is repeated to achieve the minimum travel distance, while each MDC satisfies delay and energy constraints. More specifically, let us define *b* as the number of rounds, and each round consists of *S* steps. We also define $G_{i}$ as a group with the maximum travel distance of MDC, and $G_{i-1}$ is its clockwise adjacent group. Let $MDC_{i}$ and $MDC_{i-1}$ denote the MDCs for $G_{i}$ and $G_{i-1}$, respectively. Furthermore, let us assume that $N_{i}$ and $N_{i-1}$ are sets of nodes in $G_{i}$ and $G_{i-1}$, respectively. Let *h* denote a node in $N_{i}$ that is nearest to the slant. Therefore, *h* is re-assigned from $N_{i}$ to $N_{i-1}$, and find the new path for both MDCs. This process is repeated *S* times. Among the *S* steps, NNA select the paths for MDCs that lead to minimizing the total travel distance while meeting delay and energy constraints for each MDC. Then, the next round begins, and the process is repeated until no further decrease in the total travel distance can be achieved.

**Figure 3:**
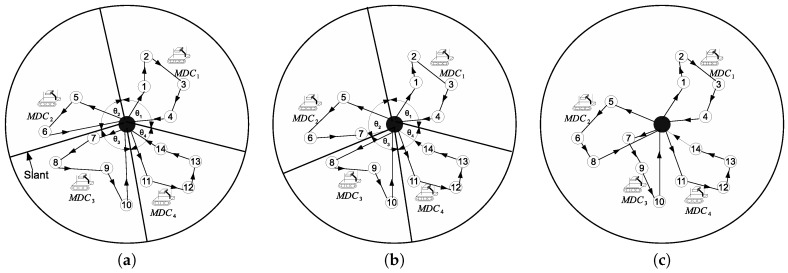
An example of group membership-adjustment algorithms: (**a**) initial route of MDCs, (**b**) nearest node assignment and (**c**) node assignment with maximal path-length decrease.

Figure 3 illustrates the node assignment process of the proposed algorithm. In the example in Figure 3a, nodes are partitioned into four groups using the clustering algorithm. In each group, an MDC is employed to collect data and recharge sensor nodes’ batteries. The path of each MDC is obtained by using the twice around the tree heuristic algorithm of TSP, and the path length is estimated. As shown in an example in Figure 3a, $MDC_{3}$ has the maximum travel distance and node set of Group 3, $N_{3}$ = {7,8,9,10}. From $N_{3}$, Node 7 is chosen for reassignment from Group 3 to Group 2, since Node 7 is nearest to the slant. Therefore, Node 7 is removed from $N_{3}$ and added to $N_{2}$, i.e., $N_{2}$ = {5,6,7} and $N_{3}$ = {8,9,10}. New paths of $MDC_{2}$ and $MDC_{3}$ are formed with the current nodes of each group, as shown in Figure 3b. The group membership-adjustment algorithm is presented in Algorithm 4 (see Appendix B for the detailed group membership-adjustment algorithm). In Algorithm 4, when NNA is used, *h* is obtained from Algorithm 5.

**Algorithm 4** Group membership-adjustment algorithm.**Input: $M,fin\Omega_{K},D,Q,P$***M*: Set of groups or MDCs▹M={1,2,...,K}$fin\Omega_{K}$: Set of nodes for *K* groups▹$fin\Omega_{K}=\left\{N_{1},N_{2},...,N_{K}\right\}$*D*: Deadline for delay*Q*: Energy capacity of MDC*P*: Minimum residual energy of sensor**Output: $R=\{R_{1},R_{2},...,R_{K}\}$**▹ Set of paths for *K* MDCs1:Initialization;2:**for** each group **do**3:    use TSP heuristics to find path of $MDC_{i}$, $R_{i}$;4:    calculate $d_{i}$, $T_{i}$, $E_{i}$, $P_{i}$;    ▹$d_{i}$: travel distance of $MDC_{i}$, $T_{i}$: travel time of $MDC_{i}$, $E_{i}$: energy consumption of $MDC_{i}$, $P_{i}$: residual energy of sensor5:**end for**6:**while** $Z_{temp}\geq Z_{cur}$ **do**7:    update $Z_{temp}$ to $Z_{cur}$ and update *R* to $R_{cur}$;8:    increment *b*;                           ▹*b* = no. of rounds9:    **while** s≤S **do**                          ▹*S* = no. of steps10:        find group *i*, which has the maximum travel distance of MDC;11:        use NNA or MPD to find node *h*, which will be re-assigned;12:        remove *h* from $N_{i}$ and add *h* to $N_{i-1}$;13:        find new $R_{i}$, $R_{i-1}$ ;14:        calculate new $d_{i}$, $T_{i}$, $E_{i}$ and $P_{i}$;15:        calculate new $d_{i-1}$, $T_{i-1}$, $E_{i-1}$ and $P_{i-1}$;16:        calculate total travel distance *Z*;17:    **end while**18:    $R_{cur}$ = set of MDCs’ paths with min(Z) and {$T_{i}\leq D$ && $E_{i}\leq Q$ && $P_{i}\geq P\}$;19:    update $Z_{cur}$ to min(Z);20:**end while**21:**return** *R* as the output.

**Algorithm 5** Nearest node assignment (NNA).1:**function** NNA($N_{i}$, slant)2:    *h* = node in $N_{i}$ nearest to slant;3:    **return** *h*4:**end function**

**Algorithm 6** Node assignment with maximal path-length decrease (MPD).
1:**function** MPD($N_{i}$, $N_{i-1}$)2:    **for** each $h\in N_{i}$ **do**3:        $tempN_{i}=\emptyset$; $tempN_{i-1}=\emptyset$;4:        $tempN_{i}=N_{i}\setminus \left\{h\right\}$; $tempN_{i-1}=N_{i-1}\cup \left\{h\right\}$; ▹ examines every node membership change from $N_{i}$ to $N_{i-1}$5:        $R_{i}$ = $tspHeuristic(tempN_{i})$;              ▹ find $R_{i}$ using $tempN_{i}$6:        $R_{i-1}$ = $tspHeuristic(tempN_{i-1})$;          ▹ find $R_{i-1}$ using $tempN_{i-1}$7:        Calculate $Y=d_{i}+d_{i-1}$;        ▹*Y*: travel distance of $MDC_{i}$ and $MDC_{i-1}$8:    **end for**9:    *h* = node in $N_{i}$ whose membership change gives min(Y);10:    **return** *h*11:**end function**

#### 5.2.2. Node Assignment with Maximal Path-Length Decrease

In NNA, from all nodes in $N_{i}$, the node that is nearest to the slant is chosen for reassignment to the new group. However, such a simple strategy may not result in the greatest travel distance reduction. Moreover, there are cases where the newly assigned node is far away from the current nodes of $G_{i-1}$. In such a case, the path length of $MDC_{i-1}$ may increase significantly. In order to address the problem of NNA algorithm, MPD first evaluates the path lengths of $MDC_{i}$ and $MDC_{i-1}$ by considering every possible node membership change from $N_{i}$ to $N_{i-1}$. Then, the node is selected from $N_{i}$, whose membership change reduces the path length of both MDCs the most.

Let us define $d_{i}$ and $d_{i-1}$ as the travel distances of $MDC_{i}$ and $MDC_{i-1}$, respectively. Furthermore, let *Y* = $d_{i}$ + $d_{i-1}$. In MPD, each node is assigned from $N_{i}$ to $N_{i-1}$ sequentially, and *Y* is estimated in every case. Let *h* denote a node in $N_{i}$ whose membership change gives the minimum *Y*. Therefore, *h* is actually reassigned to $N_{i-1}$, and the other nodes remain in $N_{i}$. As shown in the example in Figure 3c, Node 8’s membership changes from Group 3 to Group 2 since the minimum *Y* can be achieved. The rest of the steps are similar to NNA. In Algorithm 4, when MPD is used, *h* is obtained from Algorithm 6.

Now, we compute the time complexity of the group membership-adjustment algorithm for one round. In the group membership-adjustment algorithm, the TSP heuristic algorithm is applied to find the MDCs’ paths. The time complexity for finding the paths for MDCs is $O(N^{2}\log_{2}N)$. Therefore, the time complexity of the group membership-adjustment algorithm when the NNA algorithm is used can be expressed as $O(N^{2}\log_{2}N)$. Since MPD examines every assignment change from one group to another, the time complexity of the group membership-adjustment algorithm in the case of MPD is $O(N^{3}\log_{2}N)$.

## 6. Performance Study

In this section, we analyze the performance of the proposed algorithms. This paper proposes two group membership-adjustment algorithms, NNA and MPD. Furthermore, two clustering algorithms (ABC and ABC-PR) are proposed. Therefore, four combinations of the proposed algorithms are evaluated in the simulations:

- NNA with ABC
- NNA with ABC-PR
- MPD with ABC
- MPD with ABC-PR

We compare the performance of the proposed algorithms with two heuristic algorithms for VRP and VRPTW: vertex relocation (VR) [35] and generalized assignment (GA) [36]. We first present the simulation parameters and performance metrics and then discuss the performance results under various scenarios.

### 6.1. Simulation Setup

Sensor nodes are randomly deployed in a circular area with a radius of 500 m, where the sink is placed at the center of the region. Each sensor generates a data packet every two seconds and stores the packet in its buffer until an MDC collects it. When an MDC arrives at a sensor node, it will spend two seconds charging the sensor node’s battery and then collects data from this sensor. The energy transfer rate from MDC to sensor is 5 J/s [25]. Based on the study in [24], energy transfer efficiency η and the distance between MDC and sensors to be charged are assumed to be 40% and 2 m, respectively. The energy capacity of the MDC is *Q* = 50 KJ [28]. In addition, we adopt the following parameter values for the energy consumption model: γ = 2, ρ = $10(pJ/bit/m^{2})$ and $e_{T}=e_{R}=e_{S}=$ 50 nJ/bit [32]. We assume that an MDC consumes 8.27 joules to travel one meter (or 0.21 J/inch) [37]. Major simulation parameters are summarized in Table 2.

In order to obtain fair results, we performed the simulations with five different seed values and took the average value. The GNU Linear Programming Kit [38] is used to solve the ILP problem. The simulator has been built using the Java language. Three performance metrics are used to evaluate the performance of algorithms:

- total energy consumption: total energy consumed by all MDCs to finish one tour
- maximum energy consumption: the maximum energy consumed among MDCs to finish one tour
- maximum travel time: the maximum travel time among MDCs to finish one tour

### 6.2. Performance Analysis in Small-Scale Scenarios

**Figure 4:**
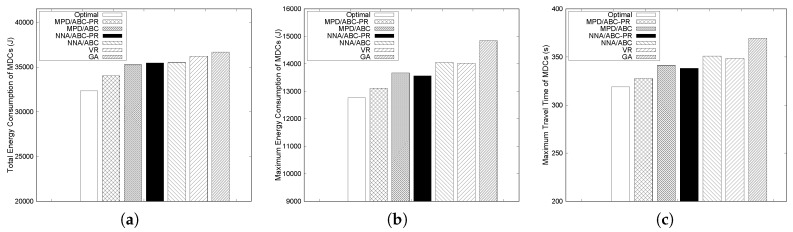
Performance of different algorithms for 15 nodes and three MDCs. (**a**) Total energy consumption of MDCs; (**b**) Maximum energy consumption of MDCs; (**c**) Maximum traveling time of MDCs.

**Figure 5:**
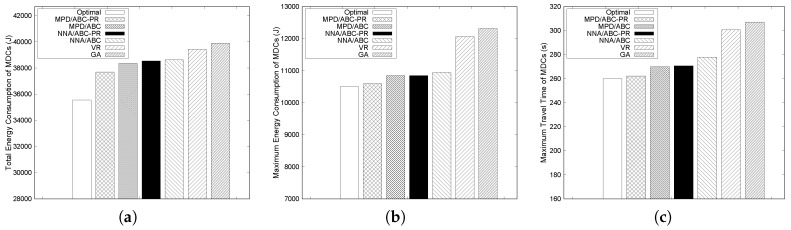
Performance of different algorithms for 15 nodes and four MDCs. (**a**) Total energy consumption of MDCs; (**b**) Maximum energy consumption of MDCs; (**c**) Maximum traveling time of MDCs.

We first compare the performance of the proposed algorithms with the optimal result obtained by solving the ILP formulation and VR [35] and GA [36] algorithms. The execution time to achieve the optimal solution increases exponentially with the number of MDCs. Thus, we consider small-scale network scenarios when comparing the performance of the proposed algorithms with the optimal solution. In this subsection, we consider 15 sensor nodes with three and four MDCs to obtain the optimal solution from the ILP formulation. Other parameters remain unchanged. Figure 4 and Figure 5 illustrate the performance of the considered schemes. The deadline for packet delay is set to 350 s and 280 s when the number of MDCs is three and four, respectively. Since MDCs collect data and deliver the collected data to the sink, each MDC needs to finish its tour within the given deadline to satisfy the delay requirement.

Figure 4a and Figure 5a show the total energy consumed by all MDCs under different schemes when the number of MDCs is three and four, respectively. The maximum energy consumption and the maximum travel time of MDCs are also presented in Figure 4 and Figure 5.

As shown in Figure 4 and Figure 5, the solution from the ILP always exhibits the lowest total energy and the lowest travel time of MDCs, since it can find the optimal paths for all MDCs. Among the proposed algorithms, MPD performs better than NNA. In particular, MPD/ABC-PR shows the best performance, except for the optimal solution. The reason is that ABC-PR estimates the path-length ratio to determine how much angular rotation is needed between two groups to balance the travel distance of the MDCs and then calculates the actual rotation angle from the path-length ratio and rotation angle. Therefore, the differences in the travel distances of MDCs tend to be small, which results in a more balanced energy consumption. As seen in Figure 4b and Figure 5b, the gap between maximum energy values with MPD/ABC-PR and the optimal solution is quite small. The maximum energy consumed by MDCs with MPD/ABC-PR is only 3% and 1% higher than the optimal solution when the number of MDCs is three and four, respectively.

For the group membership-adjustment algorithms, the group that has the maximum travel distance is chosen to reduce travel distance, and from this group, a node is reassigned to a neighboring group. MPD selects the node, such that the maximum travel distance reduction can be achieved. On the other hand, NNA selects the node that is nearest to the slant. In some cases, the newly assigned node is far from the current nodes in the group, and the travel distance of the MDCs can increase. Therefore, in Figure 4a and Figure 5a, the total energy consumption in NNA is higher than MPD. For example, when the number of MDCs is three, under ABC-PR, the total energy consumption of MPD and NNA is about 5% and 9% higher than the optimal solution, respectively.

In addition, as shown in Figure 4 and Figure 5, GA shows the highest maximum energy and travel time among the algorithms. Moreover, under GA, the total energy consumed by all MDCs is much higher than the others. Under GA, the difference between the longest and shortest paths for MDCs is high, which results in unbalanced energy consumption. Due to unbalanced paths of the MDCs, each MDC cannot satisfy the delay requirement, as shown in Figure 4c and Figure 5c. VR also shows high energy consumption, and the travel time of MDCs compared to all variants of the proposed algorithms when the number of MDCs is four and does not satisfy the delay requirement either. In both cases, all variants of our proposed algorithms can meet the delay requirement for each MDC. For example, when the number of MDCs is four, under ABC-PR, the maximum travel time of MPD and NNA is about 38 s (13%) and 30 s (10%) lower than VR, respectively.

### 6.3. Performance Analysis by Changing Network Parameters

In this subsection, we compare the performance of the proposed algorithms, VR and GA, under different numbers of MDCs, sensors and different area radii. Note that we do not evaluate the performance of the ILP solution, since it takes a large amount of time to find the solution of the ILP formulation, particularly when the number of MDCs is large. Furthermore, when we collected the results by changing network parameters, in all simulations, the proposed algorithms terminated within three rounds. Since each round consists of 20 iterations (or steps), the algorithms performed 60 iterations to find the solution for the DCEM problem.

#### 6.3.1. Effects of the Number of MDCs

**Figure 6:**
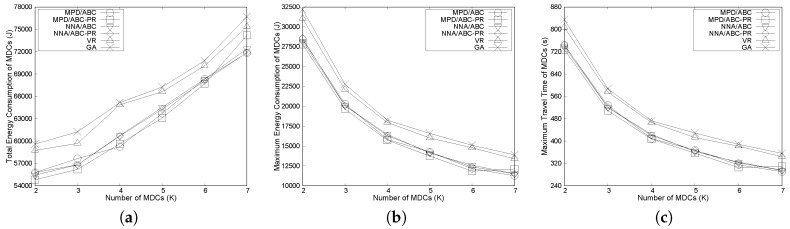
Effects of the number of MDCs: (**a**) effects on total energy consumption of MDCs; (**b**) effects on maximum energy consumption of MDCs and (**c**) effects on maximum travel time of MDCs.

We compare the network performance of the considered algorithms for different numbers of MDCs, which change from 2 to 7. As seen in Figure 6a, the total energy consumption of MDCs is higher when there are more MDCs. As shown in Figure 6a, four variants of our proposed algorithms always exhibit a lower total energy than VR and GA over different numbers of MDCs. For instance, when the number of MDCs is four, MPD/ABC-PR uses 10% and 8% less energy than GA and VR, respectively. This is because, in the proposed algorithms, nodes are partitioned into multiple groups using clustering in such a way that the travel distance of each MDC is close to the other MDCs. Moreover, in the group membership-adjustment algorithms, path length is further reduced to achieve the minimum total travel distance of MDCs while satisfying delay and energy constraints. Due to two-phase path-length balancing and minimization, MPD/ABC-PR can achieve lower energy than the others.

Figure 6b compares the maximum energy consumed among the MDCs under the algorithms. As shown in Figure 6b, the maximum energy consumed by MDCs tends to decrease when the number of MDCs increases. In addition, all variants of the proposed algorithms have lower maximum energy than VR and GA. In particular, MPD/ABC-PR consistently shows the lowest maximum energy, compared to the others. For example, when the number of MDCs is five, the maximum energy consumed among MDCs to finish one tour is about 13,700 joules under MPD/ABC-PR, compared to 16,013 joules and 16,600 joules in the cases of VR and GA, respectively. The reason is that in VR and GA, the paths for MDCs are unbalanced, and an MDC travels a long path, compared to others, which results in unbalanced energy consumption.

Figure 6c shows the maximum travel time of MDCs in the considered schemes. As shown in the Figure 6c, when the number of MDCs increases, the maximum travel time of MDCs decreases, since the distance traveled by each MDC decreases. Moreover, the two VRP heuristic algorithms always exhibit a longer maximum travel time than the others and do not satisfy the deadline for packet delay when the number of MDCs is two. In contrast, four variants of our proposed algorithms can meet the delay requirements in all cases.

#### 6.3.2. Effects of the Number of Sensors

**Figure 7:**
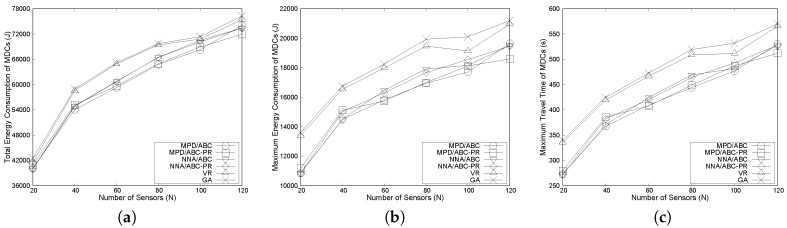
Effects of the number of sensors: (**a**) effects on total energy consumption of MDCs; (**b**) effects on maximum energy consumption of MDCs and (**c**) effects on maximum travel time of MDCs.

The effects of the number of sensors on network performance are examined for all variants of the proposed algorithm and the VR and GA algorithms. The number of sensors is set to {20, 40, 60, 80, 100, 120}, while other parameters remain unchanged. Figure 7a shows that the energy consumption of algorithms consistently increases as the number of sensors grows, since each MDC needs to visit more sensors. Moreover, as in the previous subsection, four variants of the proposed algorithms always show a lower total energy consumption than VR and GA over the variations in the number of sensors. In addition, we can see from Figure 7a that the gap between energy values of the two heuristic algorithms and our proposed algorithms is greater when the number of sensors varies between 40 and 80. For example, when the number of sensors is 60, the total energy consumption under MPD/ABC-PR is 59,653.26 joules, which is much lower than the consumed energy of GA (65,162.88 joules) and VR (64,875.77 joules).

In terms of the maximum energy consumption of MDCs, Figure 7b illustrates that the maximum energy consumed by MDCs in the considered algorithms tends to increase, and MPD/ABC-PR can achieve the lowest maximum energy consumption among the considered algorithms. This result is due to the following reasons. In ABC-PR, we balance the travel distance of MDCs as much as possible. After that, in MPD, the group having the maximum travel distance is chosen to further reduce path length. This results in the lowest energy consumption, since the MDCs consume most of their energy for movement.

As seen in Figure 7c, four variants of our algorithms always exhibit a lower maximum travel time than the other two algorithms. Furthermore, as shown in Figure 7c, when the same clustering algorithm is used, MPD has a lower maximum travel time than NNA. The reason is that, under NNA, the node that is closest to the slant is chosen for reassignment. This assignment change may not result in the lowest travel distance for MDC. In contrast, MPD first examines every assignment change and then selects the node for the actual assignment that reduces the path length by the maximum amount. Thus, MPD shows better performance compared to NNA. For example, when the number of sensors is 80, under ABC-PR, the maximum travel time of MPD and NNA is about 14% and 11% lower than GA, respectively.

#### 6.3.3. Effects of Area Radii

**Figure 8:**
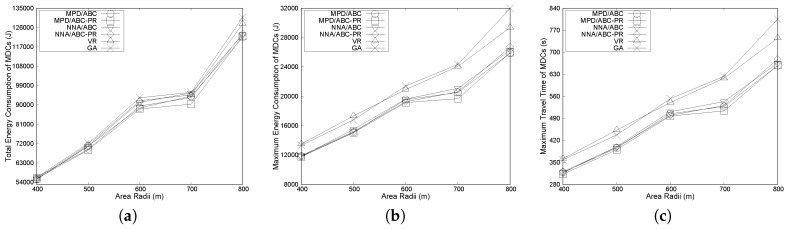
Effects of area radii: (**a**) effects on total energy consumption of MDCs; (**b**) effects on maximum energy consumption of MDCs and (**c**) effects on maximum travel time of MDCs.

In this subsection, we analyze the effects of area radii on the network performance. Radii of the circular area are varied between 400 m and 800 m. In this case, we consider the number of sensors and the number of MDCs to be 80 and five, respectively. Other parameters are kept constant. Figure 8a shows that total energy consumption rises with an increased radius. Moreover, the energy consumption of the proposed algorithm is similar to others. This result is due to the following reasons. When the same number of sensors is considered with an increased area, MPD and NNA may select similar nodes for reassignment, which results in similar energy consumption under all of the proposed algorithms.

Figure 8b,c show the variation in maximum energy and maximum travel time of the algorithms over different values for the radius. As can be seen from Figure 8b,c, the gap between the results of the two heuristic algorithms and our proposed algorithms tend to increase as area radii increase. For example, when area radius is set to 700 m, the maximum energy consumption of MPD/ABC-PR is about 4660 joules and 4400 joules lower than GA and VR, respectively. This is due to the following reasons. When the radius increases and the number of nodes is unchanged, distance among the nodes also increases, since nodes are deployed randomly in a circular area. GA and VR both only focus on minimizing total travel distance of the MDCs while finding paths for them. In contrast, our proposed algorithms find the MDCs’ paths in a balanced way with the minimum total travel distance. This leads to the increased gap between the heuristic algorithms and our proposed algorithms.

## 7. Conclusions

This paper considered a data collection scheme in which we use multiple MDCs to collect data from the sensors. In addition, MDCs also recharges the visited sensors to keep the WSN operational without the risk of energy depletion. In this paper, an optimization problem called DCEM was defined that finds the paths for MDCs in order to minimize the energy consumption considering delay and energy constraints.

We proposed the ILP optimization formulation to find the optimal solution for the DCEM problem. Furthermore, a two-phase path-selection algorithm was proposed to efficiently solve the DCEM problem. In the path-selection algorithm, clustering was performed in Phase 1 to partition the sensors into multiple groups, and in Phase 2, the membership of each group was adjusted to meet the constraints and minimize the energy consumption of MDCs. Simulations were conducted to evaluate the performance of the proposed algorithms in various scenarios. The performance of the proposed algorithms was compared with two heuristic algorithms for VRP. Simulation results showed that the proposed algorithms can achieve lower energy consumption while guaranteeing data delivery.

## Figures and Tables

**Table 1:** List of symbols. MDC, mobile data collector.

Symbol	Definition
*N*	Number of nodes
*K*	Number of MDCs
*V*	Set of nodes, V={1,…,N}
*M*	Set of MDCs, M={1,…,K}
$t_{i}$	Sojourn time of MDC at sensor *i*, (s)
$E_{i}^{ch}$	Energy transfer rate from MDC to sensor *i*, (J/s)
$E_{Q}^{k}$	Energy consumption of MDC *k* to charge visited sensors, (J)
$E_{m}$	Energy consumption of MDC to travel one meter, (J/m)
$c_{ij}$	Travel distance from node *i* to node *j*, (m)
$E_{Mv}^{k}$	Energy consumption of MDC *k* for moving, (J)
$e_{rx}$	Energy consumption to receive one bit of data, (J/bit)
*D*	Deadline of packet delay, (s)
*r*	Data generation rate of a sensor, (bits/s)
$E_{rx}^{k}$	Energy consumption of MDC *k* to receive data from visited sensors, (J)
*Q*	Energy capacity of an MDC, (J)
*v*	Speed of MDCs, (s)
η	Energy transfer efficiency
$e_{min}$	Minimum residual energy of sensors, (J)
$t_{ij}$	Travel time from node *i* to node *j*, (s)
$T_{k}$	Time for one round journey of MDC *k*, (s)
$e_{S}$	Energy consumption to generate one bit of data, (J/bit)
$e_{tx}$	Energy consumption to transmit one bit of data, (J/bit)
$u_{i}^{k}$	Number of nodes visited by MDC *k* up to node *i*

**Table 2:** Simulation parameters. Bold numbers represent default values.

Parameter	Value
Radius of circular area (*R*)	{400 **500** 600 700 800} m
Number of MDCs (*K*)	{2 3 **4** 5 6 7}
Number of sensors (*N*)	{20 40 **60** 80 100 120}
Data generation rate of sensor (*r*)	0.5 packet/s
Packet size	64 bytes or 512 bits
Speed of MDCs (*v*)	5 m/s
Number of steps (*S*)	20
Energy transfer efficiency (η)	40%
Sojourn time of MDC or charging time of sensor *i* ($t_{i}$)	2 s
Deadline of packet delay (*D*)	800 s
Energy capacity of MDC (*Q*)	50 KJ
Energy transfer rate from MDC to sensor *i* ($E_{i}^{ch}$)	5 J/s
Energy consumption of MDC to travel one meter ($E_{m}$)	8.27 J/m
Energy consumption for generating one bit of data ($e_{S}$)	50 nJ/bit
Energy usage per bit of transmission electronics ($e_{T}$)	50 nJ/bit
Energy usage per bit of reception electronics ($e_{R}$)	50 nJ/bit
